# Corticosterone Treatment and Incubation Time After Contextual Fear Conditioning Synergistically Induce Fear Memory Generalization in Neuropeptide S Receptor-Deficient Mice

**DOI:** 10.3389/fnins.2020.00128

**Published:** 2020-03-03

**Authors:** Malgorzata H. Kolodziejczyk, Markus Fendt

**Affiliations:** ^1^Neuropharmaclogy of Emotional Systems, Institute for Pharmacology and Toxicology, Otto von Guericke University Magdeburg, Magdeburg, Germany; ^2^Center for Behavioral Brain Sciences, Otto von Guericke University Magdeburg, Magdeburg, Germany

**Keywords:** neuropeptide S, memory generalization, contextual fear, corticosterone, post-traumatic stress disorder

## Abstract

Fear memory generalization is a learning mechanism that promotes flexible fear responses to novel situations. While fear generalization has adaptive value, overgeneralization of fear memory is a characteristic feature of the pathology of anxiety disorders. The neuropeptide S (NPS) receptor (NPSR) has been shown to be associated with anxiety disorders and has recently been identified as a promising target for treating anxiety disorders. Moreover, stress hormones play a role in regulating both physiological and pathological fear memories and might therefore also be involved in anxiety disorders. However, little is known about the interplay between stress hormone and the NPS system in the development of overgeneralized fear. Here, we hypothesize that NPSR-deficient mice with high corticosterone (CORT) levels during the fear memories consolidation are more prone to develop generalized fear. To address this hypothesis, NPSR-deficient mice were submitted to a contextual fear conditioning procedure. Immediately after conditioning, mice received CORT injections (2.5 or 5 mg/kg). One day and 1 month later, the mice were tested for the specificity and strength of their fear memory, their anxiety level, and their startle response. Moreover, CORT blood levels were monitored throughout the experiment. Using this protocol, a specific contextual fear memory was observed in all experimental groups, despite the 5-mg/kg CORT-treated NPSR-deficient mice. This group of mice showed a generalization of contextual fear memory and a decreased startle response, and the females of this group had significantly less body weight gain. These findings indicate that interplay between CORT and the NPS system during the consolidation of fear memories is critical for the generalization of contextual fear.

## Introduction

Dysfunctions of the brain fear circuitry can lead to anxiety or trauma stress-related disorders such as panic disorder, generalized anxiety disorder, phobias, acute stress disorder, and post-traumatic stress disorder (PTSD). One phenomenon associated with the human brain fear circuitry is the generalization of fear memory, which is an adaptive mechanism. It promotes flexible fear responses to novel situations since the fear of a past experience is generalized to be better prepared for future similar aversive situations. However, whereas generalization is adaptive, overgeneralization of fear memory is maladaptive. It can lead to a pathological state since it may induce fear behavior in inappropriate situations. Indeed, fear overgeneralization is a major feature of PTSD (Lissek et al., 2010; Kaczkurkin et al., 2017). Overgeneralization in PTSD patients is characterized by disturbances of the peritraumatic memory, which is ultimately an impairment of the specificity and strength of the fear memory. Notably, according to the diagnostic and statistical manual of mental disorders (DSM-V), PTSD patients show the clinical symptoms generally after a certain incubation time of the traumatic event. The seminal experimental approaches to model PTSD-like memory in mice focus on the generalization of fear memories that are believed to mirror the mental state in anxiety patients (Kaouane et al., 2012; Sauerhofer et al., 2012). However, the mechanisms underlying the overgeneralization of fear memory are only partly understood so far.

The neuropeptide S (NPS)/neuropeptide S receptor (NPSR) system is a neuropeptide system in the brain that may play an important role in the overgeneralization of fear memories. NPS is a 20-amino-acid peptide identified in 2002 (for review, see: Okamura and Reinscheid, 2007; Pape et al., 2010) whose structure is unique and highly conserved in the mammalian system (Reinscheid, 2007). In mice, NPS mRNA is only found in about 500 glutamatergic neurons, localized in the pericoerulear area and the Kölliker-Fuse nucleus, which both project to several forebrain areas including the limbic system (Clark et al., 2011). In these projection areas, NPSR, the only identified receptor for NPS so far (Xu et al., 2004), is widely expressed and mediates excitatory signals (for review, see: Reinscheid and Xu, 2005).

There is a growing body of literature that emphasizes the role of the NPS system in modulating arousal, stress, emotions, and cognitive functions. Various clinical studies have identified an association between polymorphisms in the NPSR gene and increased sensitivity to aversive stimuli, a higher incidence of anxiety disorders, and related behavioral endophenotypes (e.g., Raczka et al., 2010; Dannlowski et al., 2011). This concurs well with data from mice models supporting an important role of the NPS system in regulating fear and anxiety. Injections of NPS into the cerebral ventricle or into the amygdala, the central site of the brain fear circuitry, reduce conditioned fear (e.g., Jüngling et al., 2008; Fendt et al., 2010), in line with the idea that NPS-deficient mice seem to be more anxious than wild-type mice (Liu et al., 2017). Moreover, NPS injections rescue stress-induced fear extinction deficits (Chauveau et al., 2012) and boost the beneficial effects of D-cycloserine on fear extinction (Sartori et al., 2016). Interestingly, exposure to forced swim stress induces an increase of amygdaloid NPS levels (Ebner et al., 2011). All these data suggest an important role of the NPS system in the formation, consolidation, and extinction of fear memories and their modulation by stress.

Interestingly, NPSR*-*deficient mice, when bred on a C57BL*/*6J background (but see: Duangdao et al., 2009), express only a modest anxiogenic-like phenotype in behavioral paradigms of anxiety, fear, and stress (Zhu et al., 2010; Fendt et al., 2011). However, little is known about their behavioral phenotype in paradigms of pathological fear including fear generalization. A recent study from our group (Germer et al., 2019) showed no genotype effect on incubation-induced fear generalization in a one-trial contextual fear conditioning paradigm with only one retention test session. Interestingly, a significant increase in plasma CORT levels of the NPSR-deficient mice was observed at the end of the experiment. However, since the CORT levels were only measured at the end of the experiment, it is still not known whether the baseline CORT levels, the levels during the memory consolidation, or even more importantly the levels during the memory expression tests were affected by the genotype.

Several studies have shown that the release of stress hormones during and immediately after the stressful situation might influence the encoding of both physiological and pathological fear memories and thereby contribute to the development of anxiety disorders. This is supported by the observation that the hypothalamic–pituitary–adrenal (HPA) axis, responsible for regulating stress hormones, is often dysregulated in PTSD (e.g., de Kloet et al., 2005; Chrousos, 2009). Moreover, a number of studies have implicated glucocorticoid-dependent signaling underlying fear generalization (Donley et al., 2005; Kaouane et al., 2012). Interestingly, few clinical studies have identified an association between polymorphisms in the NPSR gene and increased salivary cortisol stress responses (Kumsta et al., 2013; Streit et al., 2014, 2017). This concurs well with data from mice models as the intracerebroventricular administration of NPS results in the release of corticotropin-releasing hormone (CRH), adrenocorticotropic hormone (ACTH), and CORT (Smith et al., 2006; Reinscheid, 2008). Furthermore, NPS neurons in the brainstem can be activated by CRH and thereby result in the release of NPS in brain areas related to fear circuitry such as the amygdala (Jungling et al., 2012).

The aim of the present study was to investigate whether there is an interplay between CORT and the NPS system in the development of the generalization of fear memory. We hypothesized that NPSR-deficient mice with high corticosterone (CORT) levels during the fear memories consolidation might be more prone to develop a generalization of conditioned fear. To address this hypothesis, we submitted heterozygous and homozygous NPSR-deficient mice and their wild-type littermates to a fear conditioning paradigm. In this paradigm, intense foot shocks were used and the mice received systemic injections of CORT after conditioning. Then, specificity and strength of the fear memory were examined in recent and remote retention tests in the same groups of animals to evaluate the potential development of fear memory generalization. Importantly, intense foot shocks, CORT injections, and 1-month incubation time are all factors known to induce a generalization of fear memory (Kaouane et al., 2012; Sauerhofer et al., 2012), whereas the recent memory test is able to reduce or prevent fear memory generalization (De Oliveira Alvares et al., 2013; Bueno et al., 2017). In addition to the fear memory, the startle response, anxiety-like behavior in the light–dark box, and the response to a stimulus that was presented explicitly unpaired during fear conditioning were tested. Moreover, CORT plasma levels were monitored throughout the experiment. This study shows that an interplay between CORT, NPSR deficiency, and incubation time is important in the development of generalization of fear memories.

## Materials and Methods

### Animals

Two- to three-month-old male (*n* = 90) and female (*n* = 83) wild-type (*n* = 65), heterozygous (*n* = 62), and homozygous NPSR-deficient mice (*n* = 46) from our own breeding colony (C57BL/6J background) were used and genotyped by using adequate primers (Fendt et al., 2011). Mice were housed in groups with food and water available *ad libitum*, under a 12-h light/dark cycle, and body weight was measured in the beginning and at the end of the experiment. All experiments took place during the light phase. All procedures were performed in line with the European regulations for animal experiments (2010/63/EU) and approved by the local authorities (Az. 42505-2-1172 and 1309, UniMD).

### Behavioral Studies—Apparatus and Procedure

**FIGURE 1:**
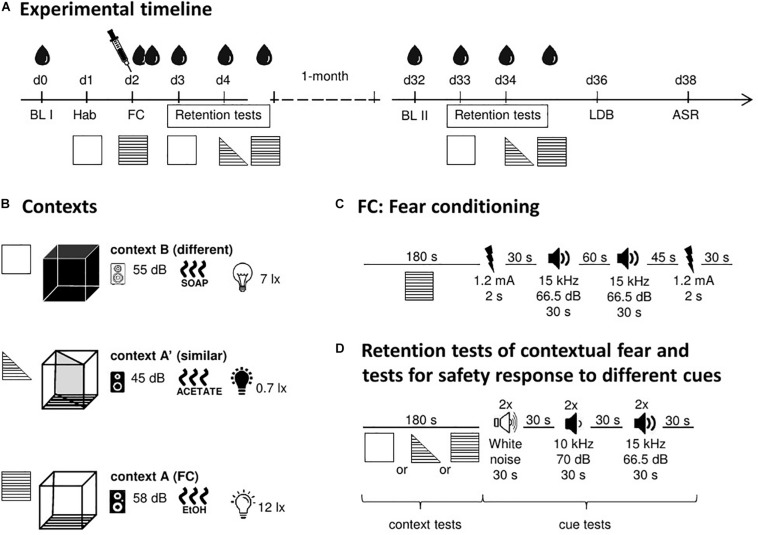
Schematic representation of the study design. **(A)** Experimental timeline with the different tests, time points for blood collections, and CORT treatment. **(B)** Details of the used contexts (background noise, odor, and illumination). **(C)** Timeline and stimuli of the fear conditioning procedure. **(D)** Timeline and stimuli of the retention tests. ASR, acoustic startle response; BL, baseline; d, day; FC, fear conditioning; Hab, habituation; LDB, light–dark box test. Symbols: blood drop, blood collection and measurement of CORT levels in plasma; syringe, injection of CORT (immediately after FC). For further details, see text.

All behavioral tests (see below) were performed with the same animals, according to the order and timelines shown in Figure 1.

#### Fear Conditioning Setup

A computerized fear conditioning system (TSE Systems, Bad Homburg, Germany) consisting of four boxes placed inside infrared sensor frames was used. Each box (46 cm × 46 cm × 32 cm) was placed in a sound-attenuating chamber provided with a loudspeaker and light sources. During the experiment (for timelines, see Figure 1A), three types of chambers were used that served to create different contexts. Context A was a square, transparent box with the floor consisting of steel grids, which were connected to a shock unit and able to deliver foot shocks of defined duration and intensity (Figure 1B). Illumination was 12 lux, the background noise was 58 dB SPL (sound pressure level), and the box was cleaned with 70% ethanol before placing the animals into it. Context A was used for fear conditioning. In addition to context A, two further contexts were used to test the specificity of the contextual fear memory. One of them [context A′ (similar)] was similar to context A and the other one [context B (different)] was clearly different. Context A′ was the same box as context A, but a diagonal divider was inserted, resulting in a triangular, transparent box. Furthermore, a lower illumination (0.7 lux) and a lower background noise (45 dB SPL) than in context A as well as 1% acetic acid as a cleaning agent were used. Context B was a square, black box with a regular floor, the brightness of 7 lux, the background noise of 55 dB SPL, and was cleaned with soapy water before each experiment. In the retention tests of the present study, the fear responses to all three chambers were tested.

Movements of animals were detected by the infrared sensors (distance: 14 mm). The freezing behavior (defined as no beam crosses for more than 1 s) was automatically recorded during all phases of the experiment. Automatically measured freezing in the TSE system is highly correlated with manual scoring (Misane et al., 2005; Endres et al., 2007).

##### Fear conditioning procedure

The timeline of the fear conditioning procedure is shown in Figure 1C. Before the experiment, animals were handled by the experimenter for 5 min for three consecutive days. On day one, all animals were individually placed into context B for 5 min (habituation). One day later, animals were fear-conditioned in context A with an unpaired cue-shock protocol (modified from Kaouane et al., 2012). Briefly, mice received the first foot shock (1.2 mA, 2 s) after 180 s, followed by an unpaired tone (T, 15 kHz, 66.5 dB SPL, 30 s) after a delay of 30 s. After an additional delay of 60 s, the same tone was presented for a second time, followed by another 45 s delay and a second foot shock. After further 30 s, mice were removed from the chamber, injected either with vehicle or one of two doses of CORT (see below) and put back in the home cages. The tones, which were presented explicitly unpaired with the foot shocks, were later used to check whether the mice erroneously associate them with the foot shocks.

##### Retention tests for contextual fear generalization and tone response

Animals were individually subjected to the retention test 24/48 h (recent retention test) and 1 month (remote retention test) after fear conditioning using the same experimental conditions. In both cases, animals were tested for their freezing behavior in context B (d3 and d33; see Figure 1A) followed by context A′ and 3 h later context A (both on d4 and d34).

In each of the contexts, the mice were placed into the box for 180 s (Figure 1D). Then, they were exposed to six acoustic stimuli (duration: 30 s) with inter-stimulus intervals of 30 s. The order of the stimuli presentation was the following: first two stimuli of white noise (N, i.e., different from the cue presented during fear conditioning) followed by two 10 kHz tones (T′, 70 dB SPL; i.e., similar to the tone used during fear conditioning) and further two presentations of tone T (tone used during fear conditioning). After a further 30 s, the mice were placed back in the home cages.

#### Systemic Injection of CORT

Immediately after fear conditioning, either vehicle (2% ethanol in saline), 2.5 mg/kg, or 5 mg/kg of CORT (Sigma-Aldrich, Taufkirchen, Germany) was injected intraperitoneally at a volume of 10 ml/kg.

#### CORT Plasma Levels

##### Repeated blood collection from the tail vein

At several time points during the experiment (see Figure 1A), blood was collected to measure plasma CORT levels. All samples were collected in the morning hours (8.00–12:00 am). The first baseline (BL) samples were collected before the habituation phase (BL I, d0). The second baseline samples were collected 1 day before the remote retention test (BL II, d32). All other samples were collected 30 min after exposing the animals to the different phases of the experiment. For blood collection, the mice were put into a Plexiglas restrainer, to which they were habituated before the experiment (5 min, 3 days). Then, the lateral tail vein was cut with a scalpel at a position 2–3 cm away from the tip of the tail (Durschlag et al., 1996). The blood was collected into EDTA-coated tubes (Microvettes, Sarstedt, Germany) and placed immediately on ice. Then, the blood samples were centrifuged (2000 rpm, 4°C for 10 min followed by collection and storage (−80°C) of plasma.

##### CORT assay

CORT levels were quantified in 100 times diluted plasma samples by an ELISA kit (Enzo Life Sciences, Lörrach, Germany). This conventional competitive ELISA was performed according to the manufacturer’s guidelines.

##### Light–dark box test

We used a system consisting of four identical boxes (49.5 cm × 49.5 cm × 41.5 cm). Each of the boxes was placed inside a frame with infrared sensors (TSE Systems, Bad Homburg, Germany). Boxes were separated into two compartments of the same size that were connected by an opening (8 cm × 6 cm). One of the compartments was dark (0.2–1.5 lux) while the other one was bright (410–570 lux).

On day 36 of the experiment (Figure 1A), mice were placed into the dark compartment and could freely explore both compartments for 10 min. Localization of the mice was measured via the infrared sensors and further processed by the TSE Phenomaster software. Between different trials, boxes were cleaned with water.

##### Acoustic startle response test

The startle response system consists of eight sound-attenuating chambers (SR-LAB, San Diego Instruments, United States). Each of the chambers (35 cm × 35 cm × 38 cm) was equipped with a loudspeaker for delivering acoustic stimuli. During the test, animals were placed into Plexiglas cylinders (4 cm **×** 10 cm) fixed onto a plate with a motion sensor underneath. Mice movements were detected by the sensor and were further analyzed by the SR-LAB software. The mean amplitude of the motion sensor output signal within 10 to 30 ms after the acoustic startle stimulus onset was used as the startle magnitude and is expressed in arbitrary units.

The acoustic startle response was measured on day 38 of the experiment (Figure 1A). After an acclimation time of 5 min, three blocks of acoustic startle stimuli (40 ms white noise) of eight different intensities (78, 84, 90, 96, 102, 108, 114, and 120 dB SPL) were presented (background noise: 60 dB SPL). Within these blocks, the order of the stimuli was pseudo-randomized. An inter-stimulus interval of 20 s was used.

### Data Analysis

To estimate the specificity and strength of the fear memory, we used the percent freezing duration (%FreD) to calculate two different indices, called context discrimination index and incubation time index. The context discrimination index was used as a measure of the specificity of contextual fear. It is defined as the ratio between the difference of freezing during exposure to two different contexts (X and Y) and the sum of the freezing in these two contexts:

$$\text{CONTEXT DISCRIMINATION INDEX}=\frac{\left(\%\text{FreD},\text{ context X})-(\%\text{FreD},\text{ context Y}\right)}{\left(\%\text{FreD},\text{ context X})+\;(\%\text{FreD},\text{ context Y}\right)}$$

A positive index means that animals express more freezing in context X than in context Y; i.e., the memory is relatively specific to context X. When the index is close to zero (means that the freezing response is equal in the two contexts) and/or have a negative value (means that animals froze more in context Y than in context X) indicates that the fear memory is not specific to a context and thereby generalized.

The incubation time index was used as a measure for the influence of incubation time (here 1 month) on the freezing in a particular context, indicating the strength of the fear memory. It is defined as the ratio between the difference of freezing during two different exposures (1st and 2nd time point) to a particular context (X) and the sum of the freezing during both exposures:

$$\text{INCUBATION TIME INDEX}=\frac{\left(\%\text{FreD},\text{ context X},\;2\text{nd time point})-(\%\text{FreD},\text{ context X},1\text{st time point}\right)}{\left(\%\text{FreD},\text{ context X},\;2\text{nd time point})+(\%\text{FreD},\text{ context X},1\text{st time point}\right)}$$

A positive index indicates an increase, zero indicates no change, and a negative value indicates a decrease of the fear memory to a particular context. Therefore, when the index is significantly more positive than zero or has a significantly more negative value, it indicates that the fear memory changed over time and thereby generalized.

We realized that the tones in the retention test decreased the freezing response of the mice, which we interpreted as a safety response. The intensity of this safety response was calculated by the following formula:

$$\begin{array}{l} \text{SAFETY RESPONSE to X}\\ \;\left(\text{stimuli T and }{\text{T}}^{\prime }\text{ or stimulus N},\text{ respectively}\right)\\ \;=(\%\text{ FreD during presentation of X})\\ \;-(\%\text{ FreD during pauses before presentation of X}) \end{array}$$

For statistical analysis, Prism 6.0 (GraphPad Software Inc., La Jolla, United States) and SYSTAT 12.0 (SPSS Inc., San Jose, United States) were used. The normal distribution of the data was checked with the D’Agostino-Pearson normality test. According to the respective experimental design, multifactorial analyses of variance (ANOVA) followed by separated one-factor or two-factor ANOVAs, if appropriate with repeated measures, were used followed by *post hoc* Holm-Sidak’s comparisons.

## Results

In all of the experiments, we tested male and female mice. In all measures except startle response (3.6.) and body weight (3.7.), a multifactorial ANOVA revealed no main effects of sex and no interactions of sex with other factors. For the measures without sex effects, we pooled sexes for further analyses and showed the pooled data in the figures.

### 5 mg/kg CORT Impaired Discrimination Between Context A and Context A′ in NPSR−/− Mice After the Incubation Time

**FIGURE 2:**
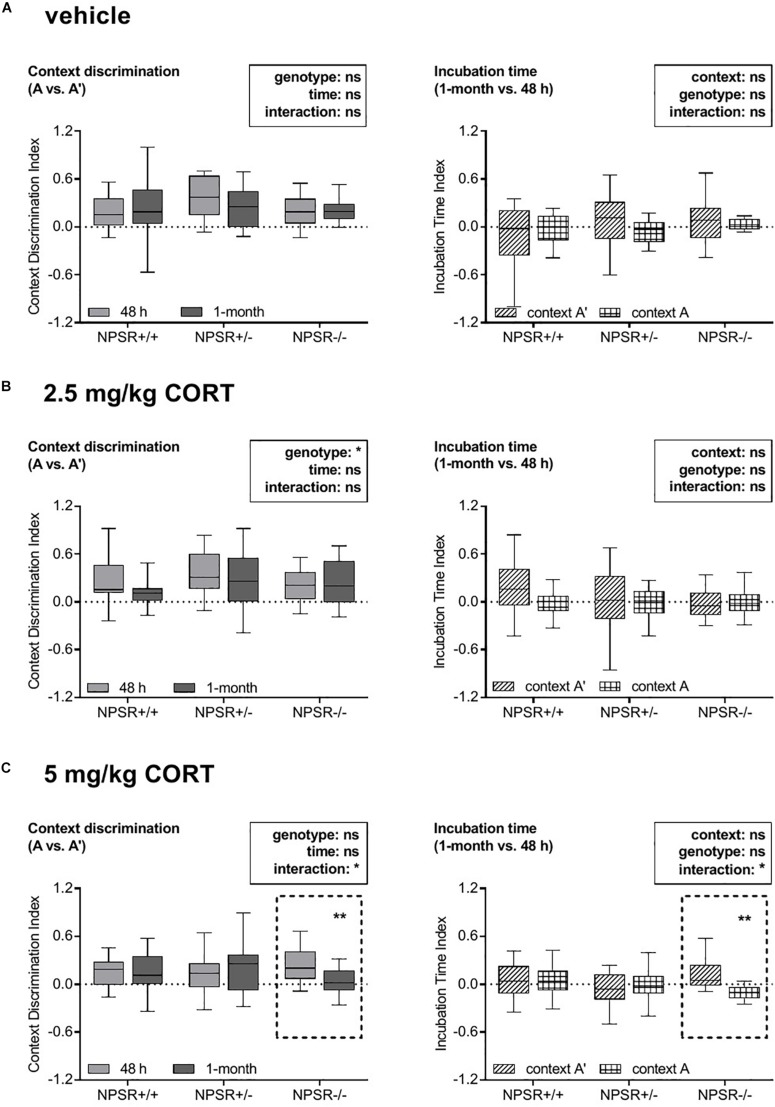
Effects of corticosterone treatment on specificity and strength of contextual fear memory in NPSR-deficient mice. The left panel depicts the context discrimination index (A vs. A′) and the right panel depicts the incubation time index (1 month vs. 48 h). Injections of **(A)** vehicle and **(B)** 2.5 mg/kg CORT did not affect specificity or strength of the contextual memory. However, injections of **(C)** 5 mg/kg CORT significantly decreased both specificity and strength of the contextual memory in NPSR−/− mice (dashed frames), suggesting a generalization of the fear memory, but not in NPSR+/+ and NPSR+/− mice. Group sizes: NPSR+/+: *n* = 65; NPSR+/−: *n* = 62; NPSR−/−: *n* = 46. Data are illustrated with box plots (median, quartiles) and Tukey’s whiskers. *p < 0.05; **p < 0.01; *post hoc* Holm-Sidak’s comparisons after ANOVA.

Our first question was whether genotype or/and treatment of the mice affected the fear responses expressed in the original fear conditioning context A, the similar context A′, and the different context B. To evaluate whether the fear responses were similar or different in these contexts, we calculated the context discrimination indices (section “Data Analysis”) for each context and each phase of the experiment. These indices were analyzed by a multifactorial ANOVA using treatment and genotype as between-subject factors and time as a within-subject factor. For the discrimination indices for contexts A and A′ (Figure 2, left panel; for original freezing data see Supplementary Figure S1), this ANOVA revealed main effects of genotype (*F*_2,164_ = 3.24, *p* = 0.04), treatment (*F*_2,164_ = 4.05, *p* = 0.02), and time (*F*_1,164_ = 4.38, *p* = 0.04), as well as an interaction between these three factors (*F*_4,164_ = 2.74, *p* = 0.03). All other interactions did not reach statistical significance. To identify the source of this interaction, we then performed ANOVAs for each treatment separately. In vehicle-treated mice, neither time nor genotype nor their interaction affected context discrimination (*F*s < 1.31, n.s.; Figure 2A, left panel). After 2.5 mg/kg CORT treatment (Figure 2B, left panel), there was an effect of genotype (*F*_2,67_ = 3.40, *p* = 0.04) but not of time and no interaction between time and genotype (*F*s < 2.56, n.s.). Notably, after treatment with 5 mg/kg CORT (Figure 2C, left panel), there was a significant interaction between genotype and time (*F*_2,164_ = 4.66, *p* = 0.01). *Post hoc* tests revealed that incubation time did not affect context discrimination in NPSR+/+ and NPSR+/− mice (*t*s < 0.14, n.s.); however, context discrimination was significantly decreased in NPSR−/− mice after 1 month (*t* = 3.24, *p* = 0.006).

Taken together, these data indicate that all treatment and genotype groups were able to discriminate between context A′ and A in the two retention tests, except the 5 mg/kg CORT-treated NPSR−/− mice, which had impaired discrimination after 1 month. The latter indicates a less specific memory, i.e., a generalization of contextual fear memory. Context A and B could always be well discriminated and no effects of time, genotype, or treatment were found (Supplementary Figure S2, left panel; for original freezing data see Supplementary Figure S3).

### 5 mg/kg CORT Impaired Contextual Fear Memory to Context A and Increased Fear to Context A′ After an Incubation Time

Next, we analyzed whether genotype or/and treatment of the mice affected the influence of incubation time on freezing behavior in contexts A, A′, and B. To address this question, we calculated the incubation time indices (section “Data Analysis”) for all contexts, treatments, and genotypes (Figure 2, right panel; for original freezing data see Supplementary Figure S1). The indices were analyzed by a multifactorial ANOVA using treatment and genotype as between-subject factors and context as a within-subject factor. This ANOVA revealed no main effects of genotype (*F*_2,164_ = 0.84, *p* = 0.43), treatment (*F*_2,164_ = 0.68, *p* = 0.51), and context (*F*_1,164_ = 1.94, *p* = 0.17), but a significant interaction between these three factors (*F*_4,164_ = 3.34, *p* = 0.01). Other interactions did not reach statistical significance (*F*s < 2.37, n.s.). Subsequently, we performed separated ANOVAs for each treatment to determine the source of this interaction. After treatment with vehicle and 2.5 mg/kg CORT, neither context nor genotype had an influence on the incubation time indices and these two factors did also not interact (*F*s < 3.05, n.s.; Figures 2A,B, right panel). However, there was a significant interaction between context and genotype in mice treated with 5 mg/kg CORT (*F*_2,53_ = 4.97, *p* = 0.01; Figure 2C, right panel). *Post hoc* comparisons revealed no influence of incubation time on the freezing behavior of NPSR+/+ and NPSR+/− mice in context A and A′ (*t*s < 0.92, n.s.). In contrast, the incubation time had significantly fewer effects on the freezing behavior of NPSR−/− mice in context A than in context A′ (*t* = 3.19, *p* = 0.007).

In sum, the effect of incubation time on freezing in context A and A′ was not affected by treatment and genotype, except in 5 mg/kg CORT-treated NPSR−/− mice. In this group, the incubation time index for context A was significantly lower than for A′, indicating an impairment of contextual fear memory after the incubation time for the original fear conditioning context and/or an increase of contextual fear in the similar context A′. This means that there is a disbalance of contextual fear to context A and A′, which suggests a contextual fear memory generalization. We also compared the incubation time index of context B with those of context A. We found no overall main effects of context, genotype, or treatment, but an effect of genotype in the group of 5 mg/kg CORT-treated mice (Supplementary Figure S2, right panel; for original freezing data see Supplementary Figure S3).

### Plasma CORT Levels

#### Plasma CORT Levels Were Enhanced After Fear Conditioning and Further Increased by Systemic CORT Injections

**FIGURE 3:**
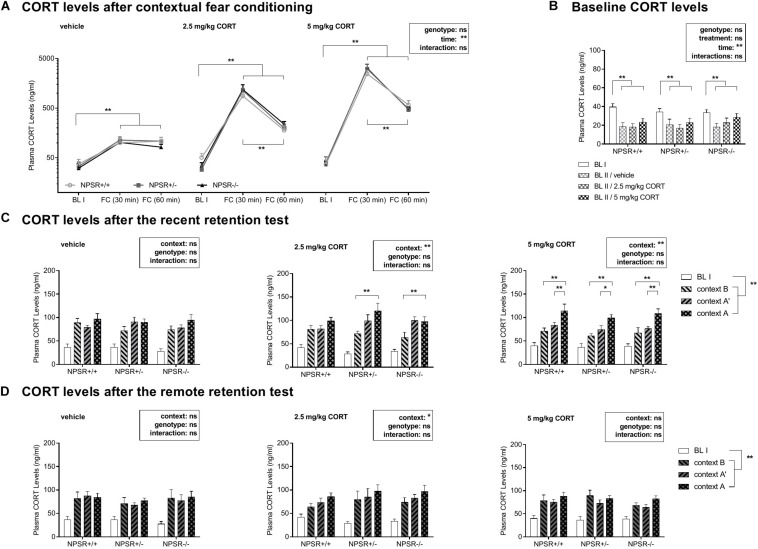
Plasma CORT levels during the study. **(A)** CORT levels are enhanced after fear conditioning and CORT injections strongly further increased these levels. **(B)** The NPSR genotype did not affect baseline CORT levels before fear conditioning. After 1 month incubation time, baseline CORT levels were decreased in all genotypes. **(C,D)** Exposure to the different contexts in the retention tests induced an increase in CORT levels. **(C)** In the recent retention tests, this increase was more pronounced in the CORT-treated NPSR+/− and NPSR−/− mice. **(D)** However, this effect was not observed in the remote retention tests. Group sizes: **(A)** NPSR+/+: *n* = 12 to 35; NPSR+/−: *n* = 15 to 41; NPSR−/−: *n* = 14 to 34. **(B)** NPSR+/+: *n* = 33 to 35; NPSR+/−: *n* = 40 to 41; NPSR−/−: *n* = 33 to 34. **(C)** NPSR+/+: *n* = 27; NPSR+/−: *n* = 27; NPSR−/−: *n* = 31. **(D)** NPSR +/+ : *n* = 27; NPSR+/−: *n* = 28; NPSR−/−: *n* = 28. Line and bar diagrams show the means + SEMs. *p < 0.05; **p < 0.01; *post hoc* Holm-Sidak’s comparisons after ANOVA.

We were wondering how plasma CORT levels change after fear conditioning and after systemic injection of CORT. Figure 3A depicts the mean plasma CORT levels in the different groups, 30 min and 60 min after fear conditioning. For analysis, a multifactorial ANOVA using treatment and genotype as between-subject factors and time as a within-subject factor was performed. There were significant main effects of treatment (*F*_2,6_ = 12.63, *p* = 0.01) and time (*F*_2,12_ = 20.56, *p* < 0.0001), as well as an interaction between these two factors (*F*_4,12_ = 12.08, *p* < 0.0001). There were neither main effects of genotype (*F*_2,6_ = 0.05, *p* = 0.96) nor significant interactions with genotype (*F*s < 0.06, n.s.). For further analysis, we performed ANOVAs for each treatment separately. In all three treatment groups, strong effects of time were found (*F*s < 23.91, *p*s < 0.0001), whereas the factor genotype or the interaction time and genotype did not reach significance (*F*s < 0.24, n.s.). Subsequent *post hoc* tests revealed a significant increase in CORT levels in all three genotypes 30 min and 60 min after fear conditioning (*F*s < 41.44, *p*s < 0.001) in relation to the first baseline CORT levels (BL I). In 2.5 mg/kg and 5 mg/kg CORT-treated mice, CORT levels declined again after 60 min (compared with 30 min; *F*s < 26.86, *p*s < 0.001). This decline was not found in vehicle-treated animals (*F* = 0.16, *p* = 0.69).

In conclusion, CORT levels were significantly increased 30 min after conditioning in all experimental groups. The intraperitoneal CORT injections immediately after conditioning strongly further enhanced CORT levels. Sixty minutes after conditioning, CORT levels were already decreased in 2.5 and 5 mg/kg CORT-treated animals. The NPSR genotype had no effects on these plasma CORT level changes and also not on baseline CORT levels (BL I) (Figure 3B).

#### CORT Treatment Increased CORT Levels After Exposure to Context A Compared With B (and A′) in the Recent Retention Test

The CORT levels after the recent and remote retention tests for contextual fear are depicted in Figures 3C,D. Separated ANOVAs with genotype as between-subject factor and context as a within-subject factor were calculated for each treatment. After the recent retention test, in vehicle-injected animals, neither context nor genotype had main effects on the CORT levels and there was no interaction (*F*s < 0.85, n.s.; Figure 3C, left panel). However, there were significant context effects after injection of 2.5 mg/kg CORT (*F*_2,44_ = 3.54, *p* = 0.0002; Figure 3C, middle panel) and 5 mg/kg CORT (*F*_2,64_ = 22.50, *p* < 0.0001; Figure 3C, right panel). *Post hoc* comparisons revealed significantly increased CORT levels after exposure to context A compared to context B in 2.5 mg/kg CORT-treated NPSR+/− (*t* = 4.09, *p* = 0.0004) and NPSR−/− mice (*t* = 3.47, *p* = 0.002). Additionally, there were increased CORT levels after exposure to context A compared to contexts B and A′ in all genotypes treated with 5 mg/kg CORT (*t*s < 3.45, *p*s < 0.03).

Taken together, 2.5 and 5 mg/kg CORT-treated mice showed increased CORT levels after exposure to context A compared with B (and A′) in the recent retention test.

#### After Incubation Time, Baseline CORT Levels Were Generally Decreased

After incubation time, baseline CORT levels (BL II) were measured again before submitting the mice to the remote retention test (Figure 3B). These levels were compared with baseline CORT levels of day 0 (BL I) and the influence of treatment and genotype as between-subject factors and time as a within-subject factor on the baseline CORT levels were examined by a multifactorial ANOVA. This analysis showed a main effect of time (*F*_1,95_ = 45.71, *p* < 0.0001) but no main effects of genotype (*F*_2,95_ = 0.34, *p* = 0.71), treatment (*F*_2,95_ = 1.83, *p* = 0.17), or interactions between these factors (*F*s < 0.38, n.s.). Separated ANOVAs for each genotype were performed after. In all genotypes, CORT levels were significantly lower after incubation time (BL II) than before fear conditioning (NPSR+/+: *F*_1,30_ = 22.04, *p* < 0.0001; NPSR+/−: *F*_1,37_ = 10.39, *p* = 0.003; NPSR−/−: *F*_1,28_ = 18.30, *p* < 0.0001). Treatment did not influence baseline CORT levels in NPSR+/+ and NPSR+/− (*F*s < 0.19, n.s.); however, there was a trend for increased CORT levels (BL II) in NPSR−/− mice (*F*_2,28_ = 2.57, *p* = 0.09). Then, when the mice were exposed to different contexts in the remote retention test (Figure 3D), the context did only have a weak effect on CORT levels. There was only a significant main effect of context in 2.5 mg/kg CORT-treated mice (*F*_2__,46_ = 3.54, *p* = 0.04).

Overall, baseline CORT levels were decreased after an incubation time of 1 month in all three genotypes. No significant difference between different genotypes or treatments was observed. In addition, the different contexts in the remote retention test did not differently affect CORT levels.

### Treatment With 2.5 and 5 mg/kg CORT Decreased Fear Inhibition by Previously Unpaired Stimuli in NPSR+/+ Mice but Not in NPSR+/− and NPSR−/− Mice

**FIGURE 4:**
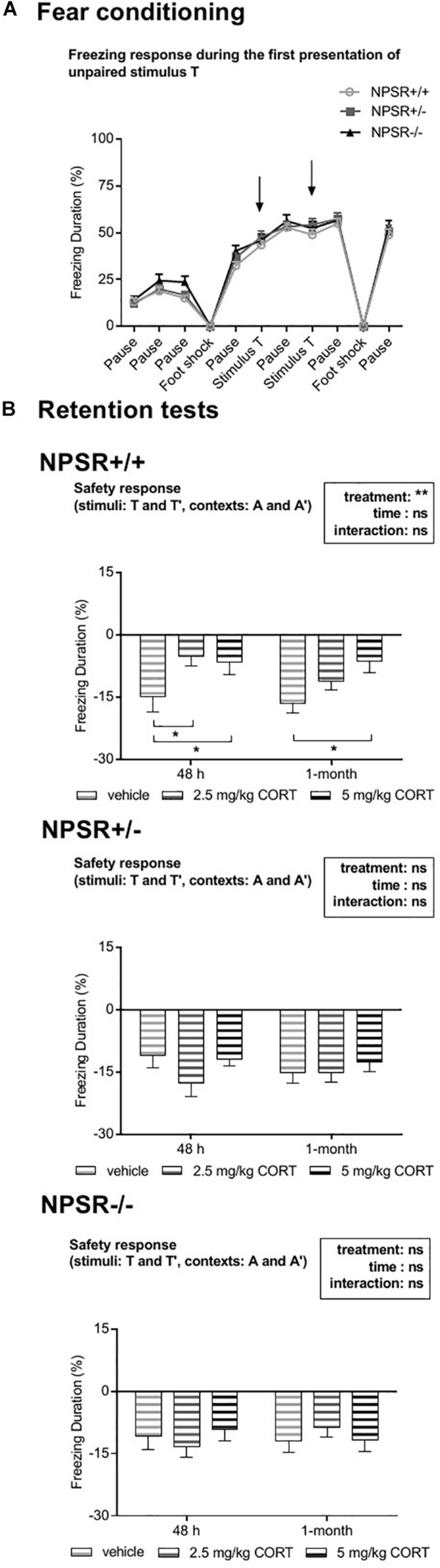
Effects of explicitly unpaired tone stimuli on contextual fear. **(A)** During fear conditioning, presentations of the tone T (arrows) did not affect freezing behavior. **(B)** Presentations of T and T′ during the different retention tests reduced contextual fear, indicating fear inhibition (i.e., a safety effect). **(B)** In CORT-treated NPSR+/+ mice, this inhibition was significantly reduced. Group sizes: NPSR +/+ : *n* = 65; NPSR+/−: *n* = 62; NPSR−/−: *n* = 46. Line and bar diagrams show the means + SEMs. *p < 0.05; *post hoc* Holm-Sidak’s comparisons after ANOVA.

During fear conditioning, also two tones T were presented explicitly unpaired to the electric foot shocks. These tones were presented to test whether such unpaired stimuli would maybe (erroneously) be associated with the aversive foot shocks and thereby later be able to induce fear. Surprisingly, presentations of this tone T and also a similar tone T′ during exposures to contexts A and A′ robustly and significantly inhibited freezing in both recent and remote retention tests (one-sample *t* tests, comparison with zero, i.e., no change in freezing: *t*s > 2.13, *p*s < 0.05). Notably, tone T had no effects on freezing during the conditioning procedure (Figure 4A). Moreover, presentations of the novel stimulus N during exposures to contexts A and A′ had also no effects in freezing inhibition (Supplementary Figure S4). This suggests that tone T was learned as a safety signal and that this memory was generalized to the similar tone T′. Importantly, all three stimuli (T, T′, and N) had no robust effects in the different context B, in which also less freezing was expressed (cf. Supplementary Figure S3). Hence, only the mean inhibition of freezing by the tones T and T′ during exposures to contexts A and A′ are depicted in Figure 4B. To analyze the safety effects of the tones, we calculated the mean difference between freezing during the tone presentations and freezing in the minute before tone presentation. Separated ANOVAs for each genotype revealed a significant treatment effect in NPSR+/+ mice (*F*_2,56_ = 5.63, *p* = 0.006; Figure 4B) but no effects of time or an interaction of time and treatment (*F*s < 1.54, n.s.). *Post hoc* comparisons with vehicle treatment showed decreased safety responses in 2.5 and 5 mg/kg CORT-treated NPSR+/+ mice in the recent retention tests (*t*s > 2.11, *p*s < 0.04) and in 5 mg/kg CORT-treated NPSR+/+ mice in the remote retention test after 1 month (*t* = 2.55, *p* = 0.02). In NPSR+/− and NPSR−/− mice, neither treatment nor time affected the difference scores, and there was no interaction (*F*s < 2.06, n.s.; Figure 4B).

Taken together, treatment with 2.5 mg/kg and 5 mg/kg CORT decreased fear inhibition by stimuli T and T′ in NPSR+/+ but not in NPSR+/− and NPSR−/− mice.

### Anxiety-Like Behavior in the Light–Dark Box Test Was Not Affected

**FIGURE 5:**
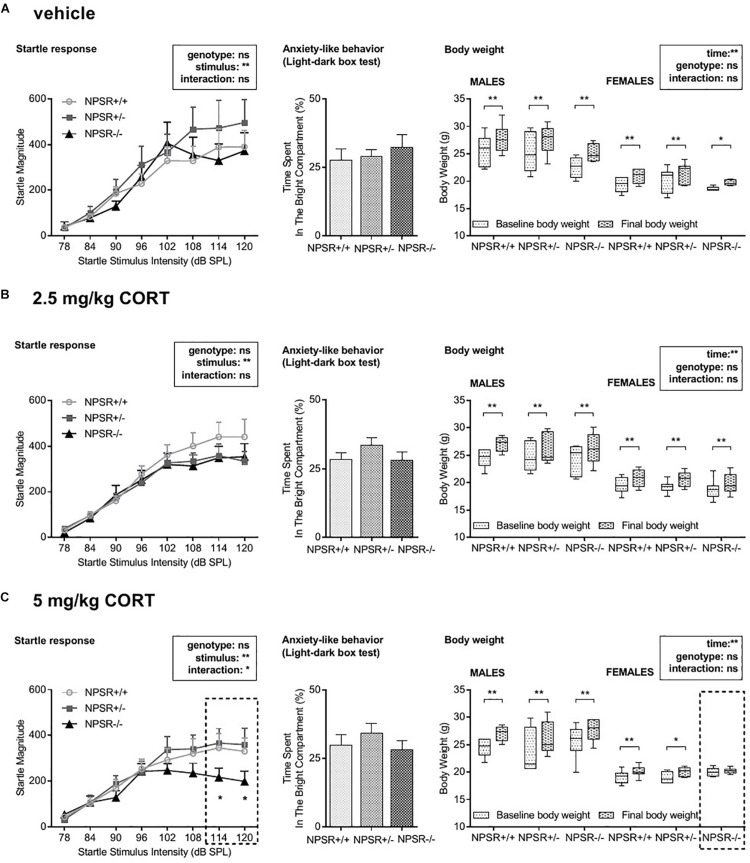
Acoustic startle magnitudes (left panel), anxiety-like behavior in the light–dark box test (middle panel), and body weight gain (right panel). **(A)** The magnitude of the acoustic startle response was generally dependent on the startle stimulus intensity. Genotype and CORT treatment had no effects except in 5 mg/kg CORT-treated NPSR−/− mice, in which the startle response to high stimulus intensities was impaired. **(B)** Neither CORT treatment nor genotype affected anxiety-like behavior in the light–dark box. **(C)** Male mice had generally more body weight gain than females. During the experiment, body weight was increased independently of genotype and treatment except for 5 mg/kg CORT-treated NPSR −/− female mice. Group sizes: NPSR +/+ : *n* = 54; NPSR+/−: *n* = 50; NPSR−/−: *n* = 40. Data are illustrated with bar and line diagrams (means + SEMs; left and middle panel) or box plots (median, quartiles) and Tukey’s whiskers (right panel). *p < 0.05; **p < 0.01; *post hoc* Holm-Sidak’s comparisons after ANOVA.

The light–dark box test was used to evaluate potential changes in anxiety-like behavior. Figure 5 (middle panel) depicts the percent time the animals spent in the bright compartment. Additionally, we analyzed the distance traveled, latency, and number of entries to the bright compartment during the test (Supplementary Figure S5). A multifactorial ANOVA using treatment, genotype, and sex as between-subject factors was used to analyze the data. The time the animals spent in the bright compartment was not affected by any of these factors. Additionally, no interactions (*F*s < 0.84, n.s.) were found, despite a significant interaction between all these three factors (*F*_4,128_ = 2.51, *p* = 0.045). Separated ANOVAs by treatment were used to identify the cause of this interaction, however, no further effects were found (*F*s < 2.41, n.s.). Analysis of the other behavioral readouts provided very similar results (Supplementary Figure S5).

Overall, anxiety-like behavior was not affected in the present study.

### 5 mg/kg CORT-Treated NPSR−/− Mice Showed Decreased Startle Responses After the Incubation Time

The acoustic startle response paradigm was chosen to evaluate potential changes in stimulus reactivity and/or arousal (Figure 5, left panel). A multifactorial ANOVA using treatment, sex, and genotype as between-subject factors and startle stimulus intensity as a within-subject factor was performed. In general, male mice had higher startle magnitudes than female mice (*F*_1__,126_ = 4.67, *p* = 0.03) but no interactions between sex and other factors were found (*F*s < 1.70, n.s.). The ANOVA further revealed a main effect of startle stimulus intensity (*F*_7,945_ = 141.57, *p* < 0.0001) but not of genotype (*F*_2,135_ = 0.80, *p* = 0.45) or treatment (*F*_2,135_ = 0.87, *p* = 0.42), and there was no interaction between these factors (*F*_4,135_ = 0.51, *p* = 0.73). This analysis was mainly confirmed by separated ANOVAs for each treatment. Startle magnitude was affected by startle (stimulus) intensity (*F*s > 14.80, *p*s < 0.0001) but not by genotype (*F*s < 2.61, n.s.). However, a significant interaction between stimulus intensity and genotype was found in 5 mg/kg CORT-treated mice (*F*_14,259_ = 1.79, *p* = 0.04; Figure 5C, left panel) but not after vehicle or 2.5 mg/kg CORT treatment (*F*s < 0.41, n.s.; Figures 5A,B, left panel). *Post hoc* tests revealed that 5 mg/kg CORT-treated NPSR−/− mice had significantly decreased startle magnitudes after stimulus intensities of 114 and 120 dB SPL.

In conclusion, after the incubation time, only 5 mg/kg CORT-treated NPSR−/− mice expressed decreased startle magnitudes at higher startle stimulus intensities.

### 5 mg/kg CORT-Treated NPSR−/− Female Mice Showed No Significant Body Weight Gain With the Incubation Time

We further tested whether the body weight of the mice was affected by sex, genotype, treatment, or time (from d0 to d39; Figure 5, right panel). We performed a multifactorial ANOVA with treatment, sex, and genotype as between-subject factors and time as a within-subject factor. There were no main effects of genotype or treatment (*F*s < 1.06, n.s.). However, there were main effects of sex (*F*_1,124_ = 256.06, *p* < 0.0001) and time (*F*_1,124_ = 298.06, *p* < 0.0001) as well as a significant interaction between these two factors (*F*_1,124_ = 24.37, *p* < 0.0001). Moreover, we found interactions of genotype and treatment (*F*_4,124_ = 2.84, *p* = 0.03), time, genotype, and treatment (*F*_4,124_ = 2.61, *p* = 0.04), and time, genotype, sex, and treatment (*F*_4,124_ = 3.11, *p* = 0.02). All other interactions did not reach statistical significance. To identify the source of these interactions, we then performed ANOVAs for each sex and treatment separately. Separated ANOVAs revealed that body weight was affected by time (*F*s > 17.63, *p*s < 0.0005) but not by genotype (*F*s < 2.13, n.s.) in all experimental groups. Additionally, a trend for an interaction between time and genotype was found in 5 mg/kg CORT-treated female mice (*F*_2,18_ = 2.50, *p* = 0.11; Figure 5C, right panel) but not after vehicle or 2.5 mg/kg CORT treatment (*F*s < 0.56, n.s.). *Post hoc* tests revealed a significant body weight gain for males and females, independent of the genotype, in all three treatment groups (*t*s > 2.84, *p*s < 0.01) but not in NPSR−/− female mice after 5 mg/kg CORT treatment (*t* = 0.53, *p* = 0.60).

Taken together, male mice generally had a higher body weight gain than females. Notably, 5 mg/kg CORT-treated NPSR−/− female mice expressed no body weight gain during this experiment.

## Discussion

The main goal of this study was to investigate the hypothesis that NPSR-deficient mice with high CORT levels during fear memory consolidation are more prone to develop generalized fear. In order to study the development of fear memory generalization, we used contextual fear conditioning and systemically injected CORT during the consolidation of fear memories. Fear memory generalization was evaluated by measuring two parameters: the strength of the fear memory and the specificity of the fear memory. To investigate the strength of the fear memory, we submitted the same group of mice to both a recent and a remote memory test, which usually improves the fear memories in the long term, i.e., it can prevent the generalization of fear memory. This allowed in some experimental groups a more specific investigation of the possible interplay between CORT and the NPS system in the development of generalized fear memory. To investigate the specificity of fear memory, we exposed the mice to three different contexts. Two of them were very similar to each other (conditioning context A and context A′) and one of them was different (context B).

In our analyses, we first examined whether incubation time, treatment, genotype, and sex affected the specificity of fear memory in different contexts, i.e., generalization of fear memory. We observed that at both experimental time points (24–48 h and 1 month after fear conditioning), all treatment and genotype groups, regardless of the sex, showed a relatively specific fear memory to the conditioning context A (Figure 2, Supplementary Figure S2, left panels; for original freezing data see Supplementary Figures S1, S3), except the group of 5 mg/kg CORT-treated NPSR−/− mice. These animals expressed very similar fear responses in the two similar contexts (A and A′) after incubation time; i.e., they did not discriminate between these two contexts any more (Figure 2C, left panel; for original freezing data see Supplementary Figure S1). This impaired specificity of fear memory indicates that a synergistic interplay of CORT treatment and NPSR deficiency interferes with the consolidation of fear memories and induces fear memory generalization after incubation time. This generalization may mirror the maladaptive state that can be measured in, e.g., PTSD patients.

We further examined whether treatment, genotype, and sex affected the strength of the fear response in a particular context with incubation time (Figure 2 and Supplementary Figure S2, right panels; for original freezing data see Supplementary Figures S1, S3). The incubation time index was not affected by treatment and genotype, regardless of the sex, except again in the group of NPSR−/− mice treated with 5 mg/kg CORT. Here, the incubation time index for context A was significantly lower than for A′, indicating an impairment of contextual fear memory after the incubation time for the original fear conditioning context and/or an increase of contextual fear in the similar context A′.

Both the decrease in the strength of fear memory after incubation time and the impaired specificity of the fear memory discussed above underlines our hypothesis. NPSR deficiency and high CORT levels during fear memory consolidation support the generalization of fear memory, here induced by incubation time. Previous studies have shown that CORT injections after fear conditioning training or incubation time alone lead to a generalization of fear memories in wild-type animals (e.g., Cordero and Sandi, 1998; Siegmund and Wotjak, 2007; Kaouane et al., 2012; Sauerhofer et al., 2012) (but see: Bueno et al., 2017). We could not observe such a generalization in our NPSR+/+ littermates and NPSR+/− mice. However, it has been shown that exposing animals to more than one retention test improves the specificity of fear memories in the long term, i.e., prevents the development of generalized fear memory (De Oliveira Alvares et al., 2013; Bueno et al., 2017). These findings explain the absence of fear generalization in our NPSR+/+ littermates and NPSR+/− mice after CORT treatment and incubation time. However, adding a further factor, i.e., NPSR deficiency, leads to fear memory generalization in our study. This clearly supports the idea that the NPS system is involved in fear memory generalization.

In addition to freezing behavior, we also measured the response of the mice to a tone stimulus (T) that was previously presented during the fear conditioning session, as well as to a similar tone T′ and a novel sound N (Figure 1D). Importantly, tone T had not predicted the unconditioned stimuli during fear conditioning but had been presented explicitly unpaired (Figure 1C). In fact, such explicitly unpaired stimuli can be learned as a safety stimulus (for review, see: Kong et al., 2014) but we did not expect such a learning, since only two tone stimuli were presented in our protocol, whereas in safety learning studies, typically much more of these explicit unpairings are presented (>12; e.g., Pollak et al., 2010a; Pollak et al., 2010b; Khalil and Fendt, 2017). Our original aim was to present these stimuli to test whether such explicitly unpaired stimuli would erroneously be associated with the unconditioned stimuli and thereby later be able to induce fear which would be a further sign of fear generalization. However, we observed robust inhibition of contextual freezing during the presentation of the tones T and T′ (Figure 4B) in the retention tests. Importantly, tone T did not affect freezing behavior during the conditioning session (Figure 4A), indicating that this fear inhibition during the retention tests was learned and most probably reflects a safety response. In NPSR+/+ mice, this effect of the tones T and T′ was reduced by CORT treatment. However, CORT treatment did not affect the tone effects in NPSR+/− and NPSR−/− mice. In human anxiety disorders, an impaired safety response has been repeatedly reported (e.g., Lohr et al., 2007; Jovanovic et al., 2009, 2012; Lissek et al., 2009; Norrholm et al., 2013; Sijbrandij et al., 2013). In the present study, CORT treatment only impaired the safety response in NPSR+/+ mice but not in NPSR+/− and NPSR−/− mice, despite the latter showing a generalization of fear memory. This indicates a beneficial effect of NPSR deficiency in learned safety (since CORT had no effects) and is in line with recent findings of our laboratory showing that NPSR-deficient mice have more pronounced safety learning (Kreutzmann et al., 2019). Of note, the observation that NPSR deficiency and CORT treatment differently interact in fear generalization and fear inhibition argues for dissociative mechanisms underlying these two phenomena.

Regarding CORT levels, dysregulations in HPA axis functioning and the connected changes in CORT levels were implicated in the development of anxiety disorders (for review, see: de Kloet et al., 2005; Chrousos, 2009). In our study, we systematically measured CORT levels throughout the different phases of the experiment with the aim to understand how changes in CORT plasma levels may be associated with the generalization of fear memories. As expected, we observed a significant increase in CORT levels 30 min after conditioning in all experimental groups (Figure 3A) showing that CORT levels robustly reflect the stress by fear conditioning. As intended, CORT injections after fear conditioning strongly enhanced the CORT levels. In the first/recent retention test, all mice injected with 5 mg/kg CORT had higher CORT levels after exposure to the conditioning context than to the similar or different context (Figure 3C, right panel). Considering 2.5 mg/kg CORT injections, this effect was only observed in NPSR+/− and NPSR−/− mice and not in the wild-type littermates. This suggests that the NPS system is involved in the modulation of CORT release during fear retention by previous CORT injections. Elevated plasma CORT levels during the consolidation of fear memories might be related to increased CORT levels in the stress response in early retention tests and NPSR deficiency seems to affect this process. Interestingly, this effect of CORT treatment was not observed in the remote retention test after 1 month of incubation (Figure 3D).

We further measured consistently lower levels of baseline CORT after 1-month incubation time (i.e., before the remote retention tests) in all genotype and treatment groups. Our observations regarding the CORT levels are in line with observations in PTSD patients showing lower baseline levels of cortisol and higher cortisol levels following exposure to trauma reminders (for review, see: Pan et al., 2018).

Several studies have shown a functional cross-regulation of the NPS system and the HPA axis. NPS is released in the basolateral amygdala upon stress (Ebner et al., 2011) and NPS injections into the ventricle increase CORT levels (Smith et al., 2006), suggesting a bidirectional interaction of the NPS system and the HPA axis. In humans, a functional variant of the NPSR1 gene has been associated with higher levels of cortisol (Streit et al., 2017). However, in the present study, there were no effects of the NPSR genotype on baseline CORT levels or on the levels after exposure to the different contexts. This is in line with previous reports showing no difference in CORT levels in NPSR−/− mice during baseline or after forced swim stress (Zhu et al., 2010) and suggests compensatory mechanisms in NPSR-deficient mice.

Moreover, we submitted animals to the light–dark box test to evaluate the innate fear of the mice (Bourin and Hascoet, 2003). We did not observe any changes in anxiety-like behavior in our experimental groups (Figure 5, middle panel; Supplementary Figure S5). Previous reports found that NPSR−/− showed more anxiety-like behavior in the light–dark box as compared to NPSR+/− mice and the wild-type littermates (e.g., Zhu et al., 2010; Germer et al., 2019). However, the present study has only investigated anxiety-like behavior at the very end of the experiment, i.e., after fear conditioning and a total of six different retention tests. Nevertheless, we were able to show that impairment of the specificity and strength of the fear memory in NPSR−/− mice did not affect their anxiety-related behavior.

In addition, we also tested the startle response of the mice. As previously reported by others (Zhu et al., 2010; Fendt et al., 2011; Germer et al., 2019), we observed higher startle magnitudes in male mice. While previous studies observed a decreased startle response in NPSR+/− and NPSR−/− mice, we did not observe such genotype effects within the vehicle or 2.5 mg/kg CORT-treated groups. Interestingly, we only observed such a decrease in the startle response in NPSR−/− mice treated with 5 mg/kg CORT (Figure 5C, left panel). In this group, startle magnitudes to higher startle stimulus intensities were significantly lower than in 5 mg/kg CORT-treated NPSR+/+ and NPSR+/− mice. Notably, abnormalities in the expression of the startle response are commonly observed in anxiety disorders (for review, see: Beck and Catuzzi, 2012). Whereas exaggerated startle magnitudes are often reported in PTSD, there are also many studies reporting blunted startle reactivity in PTSD patients. Such blunted startle reactivities are also observed in a number of animal studies using different forms of stress. Importantly, such a reduction in the startle reactivity could not be attributed to enhanced habituation to the startle stimuli (for review, see: Beck and Catuzzi, 2012). This again supports the idea that NPSR deficiency and high CORT levels during fear memory consolidation lead to behavioral changes that are similar to those of PTSD patients.

Disturbances in the body weight are often described in response to acute or chronic stress as well as to CORT treatment (for review, see: Harris, 2015; van der Valk et al., 2018). We observed a significant increase in the body weight of all mice but interestingly not in the group of NPSR−/− mice treated with 5 mg/kg CORT, i.e., the only group that expressed a generalized fear memory and decreased startle response. However, this effect was only observed in female mice. This suggests an NPS/CORT-related hormonal imbalance in the female mice of our experiment which seems to affect body weight gain.

In summary, the present study shows that a lack of NPSR gene and high CORT levels during fear memory consolidation make mice more prone to develop a fear memory generalization over time. Notably, this fear memory generalization did not affect the anxiety-related behavior of these mice but was associated with the reduced startle response magnitudes and in females with less body weight gain. Notably, NPSR+/− mice displayed the same behavioral phenotypes as the wild-type littermates indicating that one functional copy of NPSR is sufficient for normal behavior. In our view, these findings indicate a complex interplay between the NPS system, the HPA axis, and incubation time and constitute an initial step toward finding the mechanisms underlying the development of fear memory overgeneralization. Moreover, the present data may help to explain why human polymorphisms in the NPSR gene are associated with behavioral endophenotypes of overgeneralization (“catastrophizing”; Raczka et al., 2010) and thereby also with a higher probability to develop anxiety disorders (Domschke et al., 2011; Klauke et al., 2014).

## Data Availability Statement

The datasets generated for this study are available on request to the corresponding author.

## Ethics Statement

The animal study was reviewed and approved by the Landesverwaltungsamt Sachsen-Anhalt.

## Author Contributions

Both authors designed the research study, wrote the manuscript, and analyzed the data. MK performed all the experiments.

## Conflict of Interest

The authors declare that the research was conducted in the absence of any commercial or financial relationships that could be construed as a potential conflict of interest.
