# Supplementary material for: Corticosterone Treatment and Incubation Time After Contextual Fear Conditioning Synergistically Induce Fear Memory Generalization in Neuropeptide S Receptor-Deficient Mice
Source: Front Neurosci. 2020 Mar 3;14:128. doi: 10.3389/fnins.2020.00128 (PMC7081924; doi:10.3389/fnins.2020.00128)
Supplement: Supplementary file 1 [file Presentation_1.PPTX]

## Slide 1
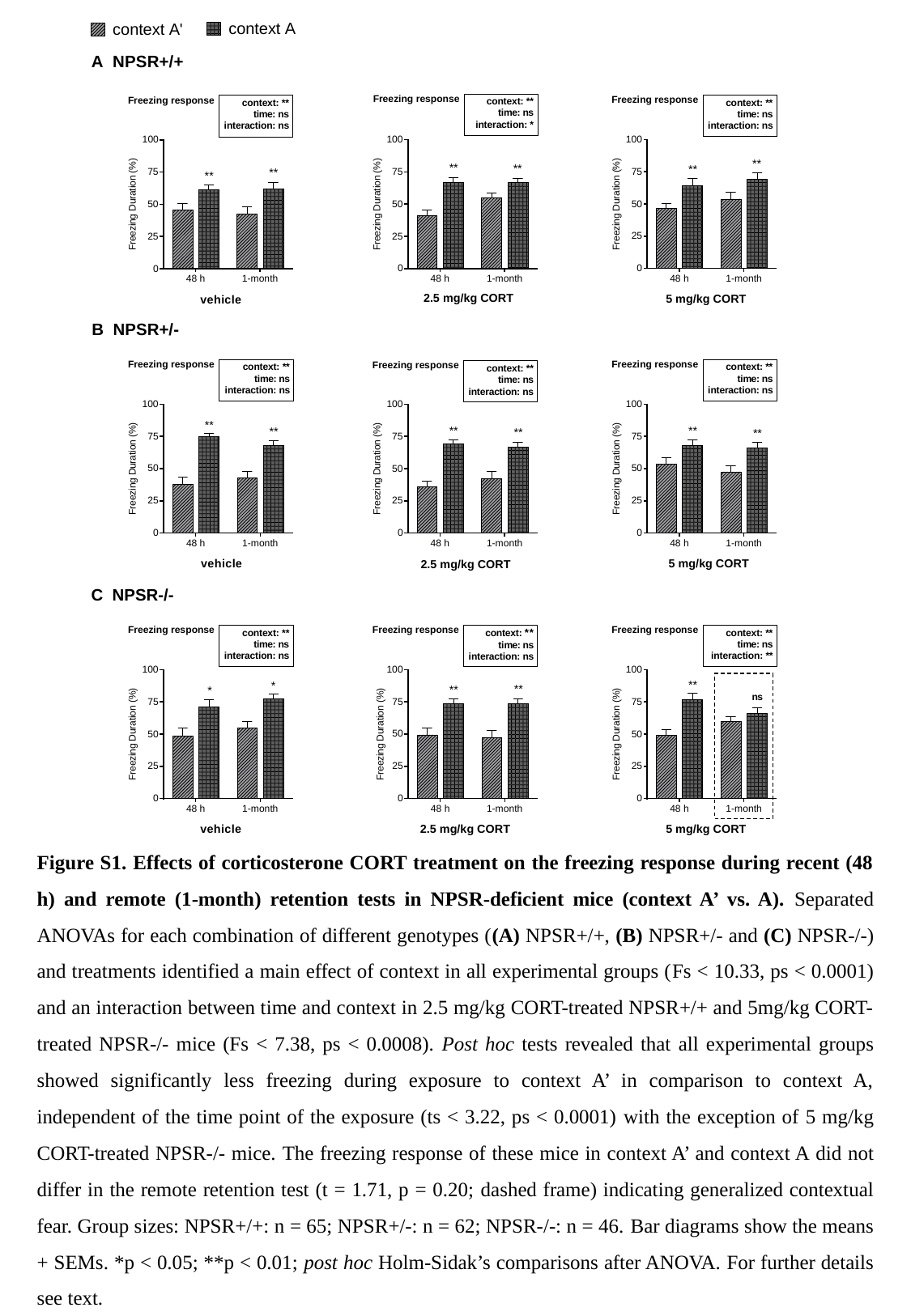

Figure S1. Effects of corticosterone CORT treatment on the freezing response during recent (48 h) and remote (1-month) retention tests in NPSR-deficient mice (context A’ vs. A). Separated ANOVAs for each combination of different genotypes ((A) NPSR+/+, (B) NPSR+/- and (C) NPSR-/-) and treatments identified a main effect of context in all experimental groups (Fs < 10.33, ps < 0.0001) and an interaction between time and context in 2.5 mg/kg CORT-treated NPSR+/+ and 5mg/kg CORT-treated NPSR-/- mice (Fs < 7.38, ps < 0.0008). Post hoc tests revealed that all experimental groups showed significantly less freezing during exposure to context A’ in comparison to context A, independent of the time point of the exposure (ts < 3.22, ps < 0.0001) with the exception of 5 mg/kg CORT-treated NPSR-/- mice. The freezing response of these mice in context A’ and context A did not differ in the remote retention test (t = 1.71, p = 0.20; dashed frame) indicating generalized contextual fear. Group sizes: NPSR+/+: n = 65; NPSR+/-: n = 62; NPSR-/-: n = 46. Bar diagrams show the means + SEMs. *p < 0.05; **p < 0.01; post hoc Holm-Sidak’s comparisons after ANOVA. For further details see text.

## Slide 2
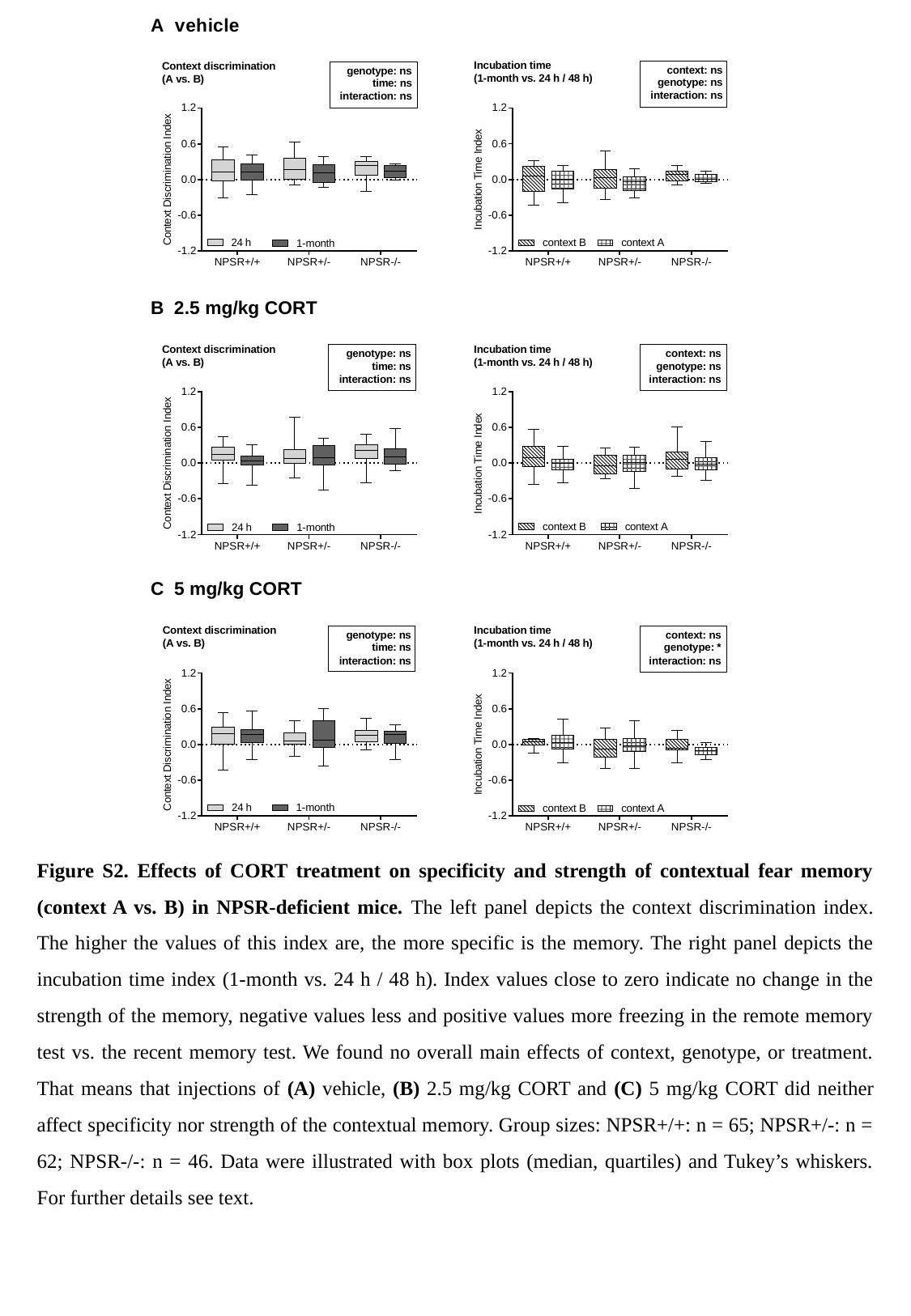

Figure S2. Effects of CORT treatment on specificity and strength of contextual fear memory (context A vs. B) in NPSR-deficient mice. The left panel depicts the context discrimination index. The higher the values of this index are, the more specific is the memory. The right panel depicts the incubation time index (1-month vs. 24 h / 48 h). Index values close to zero indicate no change in the strength of the memory, negative values less and positive values more freezing in the remote memory test vs. the recent memory test. We found no overall main effects of context, genotype, or treatment. That means that injections of (A) vehicle, (B) 2.5 mg/kg CORT and (C) 5 mg/kg CORT did neither affect specificity nor strength of the contextual memory. Group sizes: NPSR+/+: n = 65; NPSR+/-: n = 62; NPSR-/-: n = 46. Data were illustrated with box plots (median, quartiles) and Tukey’s whiskers. For further details see text.

## Slide 3
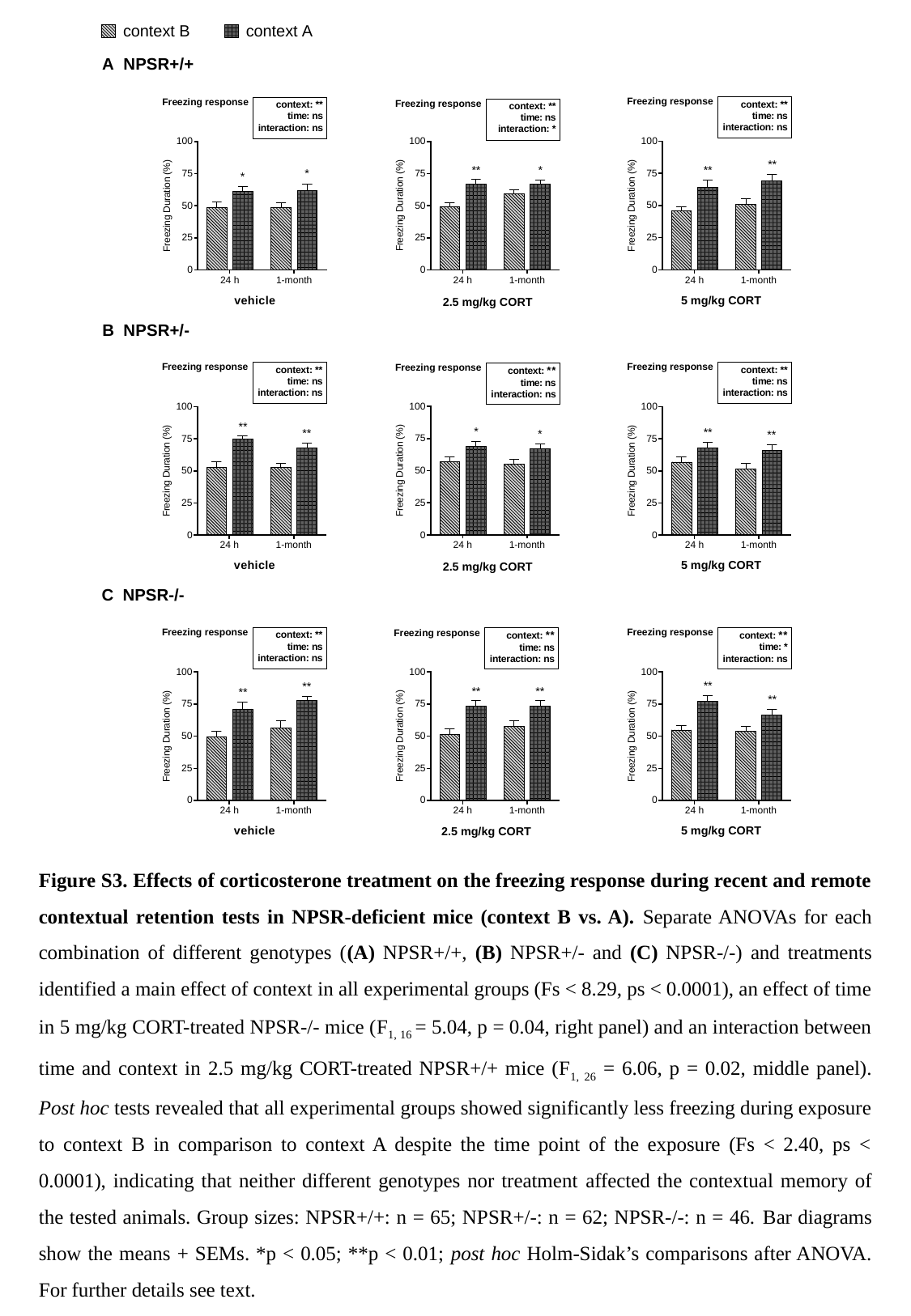

Figure S3. Effects of corticosterone treatment on the freezing response during recent and remote contextual retention tests in NPSR-deficient mice (context B vs. A). Separate ANOVAs for each combination of different genotypes ((A) NPSR+/+, (B) NPSR+/- and (C) NPSR-/-) and treatments identified a main effect of context in all experimental groups (Fs < 8.29, ps < 0.0001), an effect of time in 5 mg/kg CORT-treated NPSR-/- mice (F1, 16 = 5.04, p = 0.04, right panel) and an interaction between time and context in 2.5 mg/kg CORT-treated NPSR+/+ mice (F1, 26 = 6.06, p = 0.02, middle panel). Post hoc tests revealed that all experimental groups showed significantly less freezing during exposure to context B in comparison to context A despite the time point of the exposure (Fs < 2.40, ps < 0.0001), indicating that neither different genotypes nor treatment affected the contextual memory of the tested animals. Group sizes: NPSR+/+: n = 65; NPSR+/-: n = 62; NPSR-/-: n = 46. Bar diagrams show the means + SEMs. *p < 0.05; **p < 0.01; post hoc Holm-Sidak’s comparisons after ANOVA. For further details see text.

## Slide 4
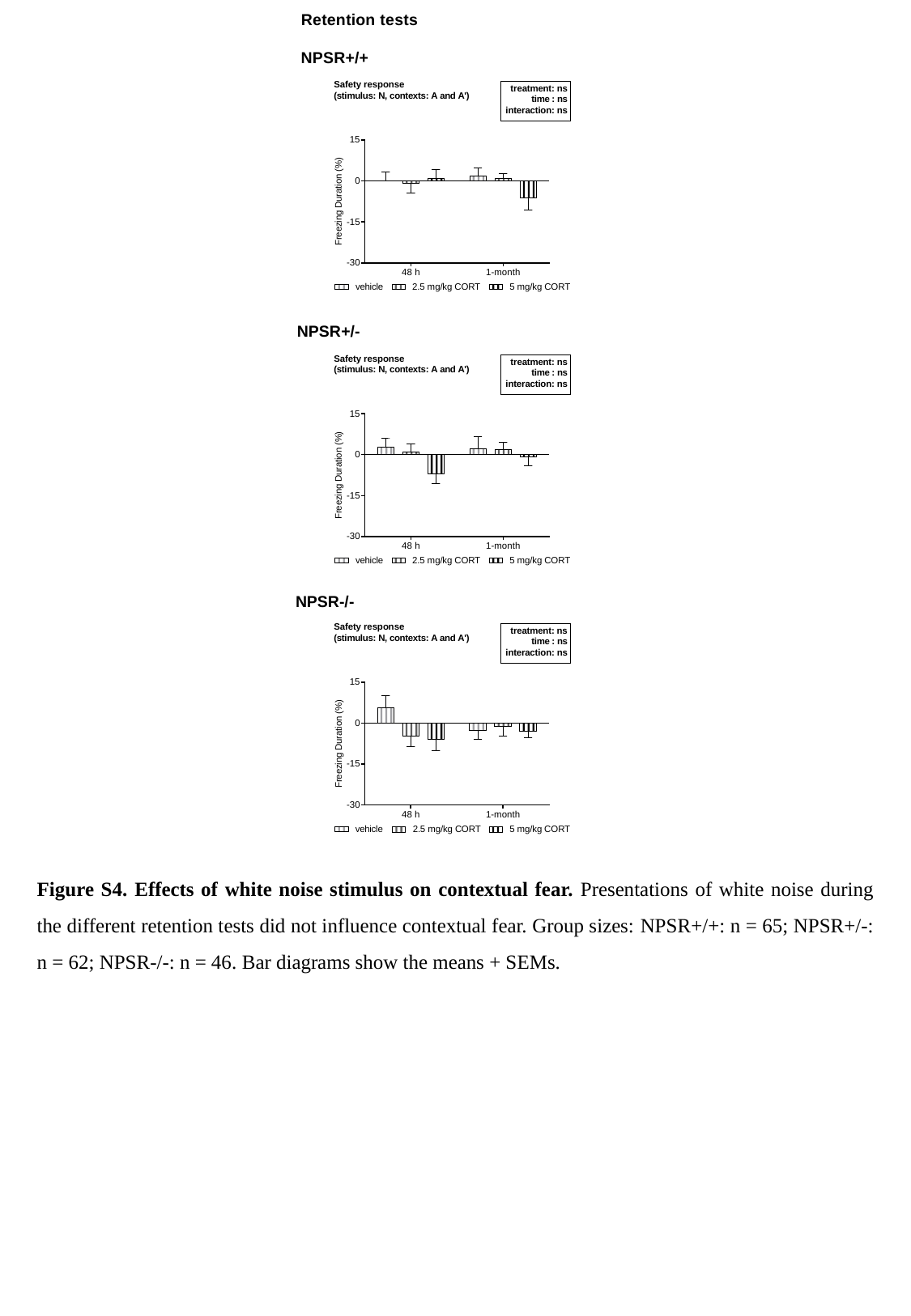

Figure S4. Effects of white noise stimulus on contextual fear. Presentations of white noise during the different retention tests did not influence contextual fear. Group sizes: NPSR+/+: n = 65; NPSR+/-: n = 62; NPSR-/-: n = 46. Bar diagrams show the means + SEMs.

## Slide 5
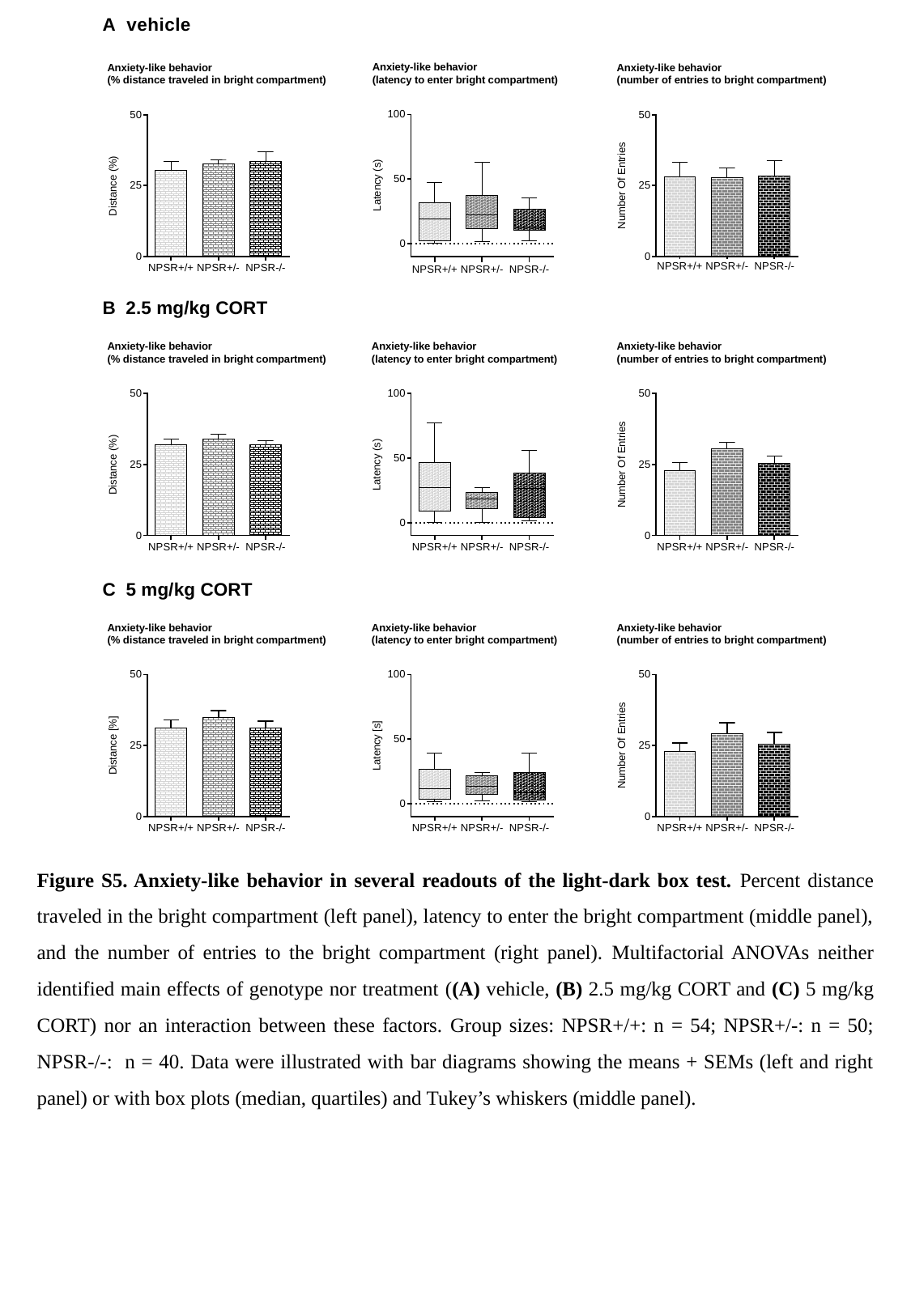

Figure S5. Anxiety-like behavior in several readouts of the light-dark box test. Percent distance traveled in the bright compartment (left panel), latency to enter the bright compartment (middle panel), and the number of entries to the bright compartment (right panel). Multifactorial ANOVAs neither identified main effects of genotype nor treatment ((A) vehicle, (B) 2.5 mg/kg CORT and (C) 5 mg/kg CORT) nor an interaction between these factors. Group sizes: NPSR+/+: n = 54; NPSR+/-: n = 50; NPSR-/-: n = 40. Data were illustrated with bar diagrams showing the means + SEMs (left and right panel) or with box plots (median, quartiles) and Tukey’s whiskers (middle panel).
